# Atherosclerosis and Autoimmunity

**DOI:** 10.1155/2018/6730421

**Published:** 2018-03-29

**Authors:** Francesca Romana Spinelli, Francesca Barone, Fabio Cacciapaglia, Arbi Pecani, Matteo Piga

**Affiliations:** ^1^Sapienza Università di Roma, Rome, Italy; ^2^University of Birmingham, Birmingham, UK; ^3^Azienda Ospedaliera Policlinico di Bari, Bari, Italy; ^4^University Hospital Center “Shefqet Ndroqi”, Tirana, Albania; ^5^Università di Cagliari, Cagliari, Italy

This special issue brings together original research and review articles focusing on the contribution of innate and adaptive immune response in the endothelial dysfunction and in the progression of atherosclerosis in autoimmune rheumatic diseases (AIRDs). By pointing out the relationship between immune response and atherosclerosis, it opens a window of opportunity for therapeutic intervention in the management of cardiovascular comorbidity.

AIRDs have been linked to a high risk of cardiovascular (CV) morbidity and mortality, mainly due to premature atherosclerosis (ATS). Recently, more attention has been attributed to ATS as an inflammatory, immune-mediated disease leading to premature vascular damage; mounting evidence supports an independent role for both humoral and cellular immune response, together with the involvement of innate immunity, in the development of the atherosclerotic plaque [1]. The immune system contributes mainly to endothelial dysfunction (ED), the earliest and reversible stage of ATS [2]. Recent evidence supports the presence of premature, accelerated ATS in AIRD patients that cannot be fully explained by the traditional cardiovascular risk factors. In this special issue, Z. Wang et al. reviewed the emerging role of the interplay between cigarette smoking and adipose tissue in the progression of atherosclerotic plaque, describing how the exposure to chemicals affects the status and functions of adipocytes.

Rheumatoid arthritis (RA) and ATS share many common mechanisms responsible for local and systemic inflammation [3]. In their original paper, G. L. Erre et al. evaluated the microvascular endothelial response in a large cohort of RA patients without any previous CV event and detected peripheral microvascular ED in one-third of the patients, which was only partially related to traditional cardiovascular risk factors. In their review of the literature, M. Di Franco et al. addressed the methylarginine metabolism in RA patients; in particular, the authors focused on asymmetric dimethyl-arginine (ADMA) and its implication in the accelerated atherosclerosis both as a surrogate biomarker of ED and as a possible target of treatment.

Immune-mediated mechanisms seem to interplay and converge into a proinflammatory and proatherogenic phenotype. In this regard, Y. Jeng et al. explored how the abundance of CD16+ monocyte subset predicts mortality: in a longitudinal cohort study, the authors showed that higher number of CD16+ monocytes correlates with increased mortality (overall mortality and CV deaths) in hemodialysis patients, shedding light on early recognition of immune dysfunction in this context.

The metabolic effects linking atherosclerosis, autoimmunity, and chronic inflammation are intriguing and still not well explored [4]. S. Gao et al. created a transgenic rabbit model that successfully expressed liver-specific human cholesteryl ester transfer protein (hCETP) with the ability to enhance macrophage-derived foam cell formation and increasing HDL cholesterol and triglyceride plasmatic levels; the authors reported an atherogenic effect of increased CETP activity during cholesterol-fed diet, supporting a role for CETP inhibition as a cardioprotective intervention. In the light of these results, the immune system interference with CETP activity should be evaluated in human clinical trials and considered for therapeutic strategies.

Through this special issue, we also provide new scientific evidence on the potential effects that medications used to treat AIRDs may have on the endothelial function and progression of atherosclerosis. *In vitro* experiments from K. Lakota et al. showed that both methotrexate and fluvastatin are highly effective in lowering proatherogenic cytokines. Such an evidence may partly explain the protective effect of methotrexate and hydroxychloroquine against accelerated ATS in patients with RA and systemic lupus erythematosus as reviewed by A. A. Mangoni et al. and by A. Floris et al., respectively. Moreover, the effects of methotrexate and TNF-inhibitors on ADMA serum levels are reviewed in the paper by M. Di Franco and colleagues.

In this special issue, we emphasized some important topics concerning the contribution of the immune system in the development of ATS in AIRDs, thus shedding light not only on the understanding of these mechanisms but also on their practical implications.

*Francesca Romana Spinelli* *Francesca Barone* *Fabio Cacciapaglia* *Arbi Pecani* *Matteo Piga*
